# Multi-Mycotoxin Occurrence in Dairy Cattle and Poultry Feeds and Feed Ingredients from Machakos Town, Kenya

**DOI:** 10.3390/toxins12120762

**Published:** 2020-12-03

**Authors:** David Chebutia Kemboi, Phillis E. Ochieng, Gunther Antonissen, Siska Croubels, Marie-Louise Scippo, Sheila Okoth, Erastus K. Kangethe, Johannes Faas, Barbara Doupovec, Johanna F. Lindahl, James K. Gathumbi

**Affiliations:** 1 Department of Veterinary Pathology, Microbiology and Parasitology, Faculty of Veterinary Medicine, University of Nairobi. P.O. Box 29053, Nairobi 00100, Kenya; kemboidc@gmail.com; 2 Department of Animal Science, Chuka University, P.O. Box 109-00625, Chuka 00625, Kenya; 3 Department of Pharmacology Toxicology and Biochemistry, Faculty of Veterinary Medicine, Ghent University, Salisburylaan 133, 9820 Merelbeke, Belgium; Gunther.Antonissen@UGent.be (G.A.); phillisemelda.ochieng@UGent.be (P.E.O.); Siska.Croubels@UGent.be (S.C.); 4 Department of Food Sciences, Faculty of Veterinary Medicine, University of Liège, Avenue de Cureghem 10, 4000 Liège, Belgium; mlscippo@ulg.ac.be; 5 Department of Pathology, Bacteriology and Avian Diseases, Faculty of Veterinary Medicine, Ghent University, Salisburylaan 133, 9820 Merelbeke, Belgium; 6 School of Biological Sciences, University of Nairobi, P.O. Box 30197, Nairobi 00100, Kenya; sheilaokoth@uonbi.ac.ke; 7 Independent Researcher, P.O. Box 34405, Nairobi 00100, Kenya; mburiajudith@gmail.com; 8 BIOMIN Research Center, Technopark 1, 3430 Tulln, Austria; johannes.faas@biomin.net (J.F.); barbara.doupovec@biomin.net (B.D.); 9 International Livestock Research Institute (ILRI), P.O. Box 30709, Nairobi 00100, Kenya; 10 Department of Medical Biochemistry and Microbiology, Uppsala University, SE-751 05 Uppsala, Sweden; 11 Department of Clinical Sciences, Swedish University of Agricultural Sciences, SE-750 07 Uppsala, Sweden

**Keywords:** aflatoxins, ergot alkaloids, feed safety, food safety, mycotoxins, sub-Saharan Africa

## Abstract

Mycotoxins are common in grains in sub-Saharan Africa and negatively impact human and animal health and production. This study assessed occurrences of mycotoxins, some plant, and bacterial metabolites in 16 dairy and 27 poultry feeds, and 24 feed ingredients from Machakos town, Kenya, in February and August 2019. We analyzed the samples using a validated multi-toxin liquid chromatography-tandem mass spectrometry method. A total of 153 mycotoxins, plant, and bacterial toxins, were detected in the samples. All the samples were co-contaminated with 21 to 116 different mycotoxins and/or metabolites. The commonly occurring and EU regulated mycotoxins reported were; aflatoxins (AFs) (70%; range 0.2–318.5 μg/kg), deoxynivalenol (82%; range 22.2–1037 μg/kg), ergot alkaloids (70%; range 0.4–285.7 μg/kg), fumonisins (90%; range 32.4–14,346 μg/kg), HT-2 toxin (3%; range 11.9–13.8 μg/kg), ochratoxin A (24%; range 1.1–24.3 μg/kg), T-2 toxin (4%; range 2.7–5.2 μg/kg) and zearalenone (94%; range 0.3–910.4 μg/kg). Other unregulated emerging mycotoxins and metabolites including *Alternaria* toxins, *Aspergillus* toxins, bacterial metabolites, cytochalasins, depsipeptides, *Fusarium* metabolites, metabolites from other fungi, *Penicillium* toxins, phytoestrogens, plant metabolites, and unspecific metabolites were also detected at varying levels. Except for total AFs, where the average contamination level was above the EU regulatory limit, all the other mycotoxins detected had average contamination levels below the limits. Ninety-six percent of all the samples were contaminated with more than one of the EU regulated mycotoxins. These co-occurrences may cause synergistic and additive health effects thereby hindering the growth of the Kenyan livestock sector.

## 1. Introduction

Mycotoxins are secondary metabolites produced by fungi and pose a serious problem to human and animal health when consumed in food and feed. These metabolites are produced by molds of different genera, in particular *Aspergillus, Fusarium*, and *Penicillium,* but also *Alternaria* and *Claviceps*. In the livestock sector, mycotoxins cause reduced feed intake and feed utilization, suppression of immunity, alter reproduction as well as causing hepatotoxicity, nephrotoxicity, mortality, and subsequently serious economic losses [1,2]. The animal health effects vary from one animal species to the other, the type of mycotoxins, duration, and levels of exposure [3]. In addition, some mycotoxins are passed into animal products such as milk, meat, and eggs and thus pose a food safety concern to humans [4,5,6,7,8,9]. In Kenya high levels of mycotoxins, especially aflatoxins (AFs) have been reported in feeds [10,11,12,13,14]. Overall, AFs are the most commonly tested and detected mycotoxins in Africa because of their high toxicity and prevalence in feed and feed ingredients. They are also the most regulated in feeds and food in many countries [3]. In Kenya, apart from AFs, deoxynivalenol (DON), fumonisins (FUM, expressed as the sum of fumonisin B1 (FB1) and fumonisin B2 (FB2)), ochratoxin A (OTA), and zearalenone (ZEN) have also been reported in animal feeds [11,13]. Regulatory limits have been set for AFs in animal feed and milk in Kenya, but not for other mycotoxins, hence there is little monitoring done for the other mycotoxins in animal feeds. This lack of regulation is also present in most sub-Saharan countries with the regulations only addressing AFs, except for South Africa where guidance levels exist for ZEN, FUM, and DON in animal feeds [15]. Worldwide, the World Health Organization/Food and Agriculture Organization of the United Nations (WHO/FAO) through the Codex Alimentarius Commission (CODEX) have set up a regulatory limit for AFB1 in animal feeds which most African countries have adopted while the European Union (EU) and the United States of America through the United States Food and Drug Agency (USFDA) have also established a regulatory limit for AFs and guidance limits for other mycotoxins [15]. And despite their regulation being stricter, the EU is a major destination of trade for most African countries, and hence the EU regulatory and guidance values are used for comparison since they may negatively impact trade and in addition they cover a wide variety of feeds for different species.

Little has been done to detect other unregulated fungal metabolites, plant toxins, and bacterial metabolites in feeds and feed ingredients in Kenya, however, they do occur in feeds with either adverse, beneficial, or unknown effects on animal health [16,17,18,19,20,21]. Ergot alkaloids are produced by fungi from the genus *Claviceps* and frequently contaminate cereals. Consumption of ergot alkaloids in feed has a negative impact on the feed intake, animal growth, and reproduction, hence affecting animal performance [19]. Other unregulated metabolites from *Aspergillus, Fusarium,* and *Penicillium* fungi have also been reported to contaminate feed with studies showing some as emerging mycotoxins having a negative impact on animal health and performance [18,20], and with some having additive effects on other regulated mycotoxins [18]. *Alternaria* mycotoxins are a group of toxins produced by fungi from the genus *Alternaria* that affect plants such as cereals and oilseeds. There are more than 70 *Alternaria* toxins that belong to the chemical groups such as nitrogen-containing compounds, steroids, terpenoids, pyranones, quinines, and phenolics with alternariol (AOH), alternariol monomethyl ether (AME), tenuazonic acid (TEA), and tentoxin (TEN) being the major and most studied and having toxicological concern [18,20]. Despite little being known on the toxicological mechanism of most *Alternaria* toxins, they are hazardous to animal health through cytotoxicity, genotoxicity, fetotoxicity, and teratogenicity [16].

Bacterial metabolites are byproducts from bacteria that contaminate feed and while they may be considered beneficial since some are antibiotics, they may also lead to increased development of antibiotic-resistant bacteria [20].

Apart from fungal and bacterial metabolites, some plant compounds found in the feed may also have adverse effects on the animal. Phytoestrogens are non-steroidal phenolic plant compounds with a similar structure to estradiol and hence bind with estrogen receptors and may inhibit or promote estrogenic response. Soybean is the major source of these phytoestrogens with dietary phytoestrogens having adverse effects on animals [21,22,23].

Co-occurrence of different mycotoxins can cause synergistic, additive, or antagonistic effects, with for example FUM reported to increase the uptake of AFs and subsequently the carry-over to milk [24]. Therefore, there is a need to regularly monitor the levels of multiple mycotoxins as well as other bacterial metabolites and plant compounds in animal feeds, to have adequate information for effective mycotoxin management and to safeguard animal and human health.

The objective of this study was therefore to assess the natural co-occurrence and levels of fungal metabolites, bacterial metabolites, and plant toxins in dairy cattle, poultry feeds, and feed ingredients used for animal feed in Kenya.

## 2. Results

**Figure 1:**
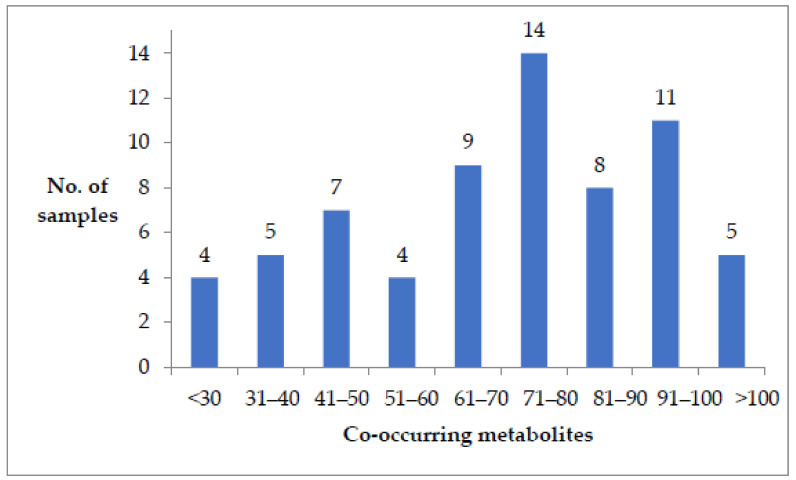
Number of samples co-contaminated with a given range of metabolites.

A total of 153 toxins, comprising mycotoxins, plant, and bacterial toxins, were detected in the samples. All the samples were co-contaminated with between 21 to 116 different mycotoxins and/or fungal metabolites (Figure 1). Further details of the co-occurrence can be found in Supplementary Table S1.

The majority of the samples (96%) contained more than one of the ten common EU regulated mycotoxins analyzed for, with 73% having 5 or more mycotoxins and 13% having 8 out of the 10 common EU regulated mycotoxins. Of the samples that were contaminated with AFs, 100% were also contaminated with ZEN, 98% had FUM, 92% had nivalenol (NIV), 89% had DON, 87% had DON-3-glucoside (DON-3-gluc), 70% had ergot alkaloids, 6% had T-2 toxin (T-2), and 4% had HT-2 toxin (HT-2). Of the feeds contaminated with fumonisin B1 (FB1), 25% also had OTA.

The most commonly occurring and EU-regulated mycotoxins, in the different types of feed and feed raw ingredients, are presented in Table 1. *Fusarium* mycotoxins; ZEN (94%; range 0.3–910.4 μg/kg), FUM (90%; range 32.4–11,658.7 μg/kg), DON (82%; range 22.2–1037 μg/kg) and NIV (73%; range 9.9–144 μg/kg) had the highest occurrence with AFs (70%; range 0.2–318.5 μg/kg), and ergot alkaloids (70%; range 0.4–285.7 μg/kg) also having a high occurrence. OTA (24%; range 1.1—24.3 μg/kg), T-2 (4%; range 2.7–5.2 μg/kg), and HT-2 (2%; range 11.9–13.8 μg/kg) occurred at a lower incidence and level.

Aflatoxin B1 (AFB1) was the most prevalent amongst the AFs contaminating 69% (range 0.5–134 μg/kg) of all the samples. The other detected AFs were AFG1 (58%; range 0.2–123 μg/kg), AFB2 (45%; range 0.4–22.1 μg/kg), AFG2 (31%; range 0.5–28.5 μg/kg) and AFM1 (22%; range 0.4–11 μg/kg).

FB1 (90%; range 32.4–8345.6 μg/kg) was the most prevalent FUM. Other FUMs were, in descending prevalence, fumonisin B2 (FB2) (85%; range 16.7–3313.1 μg/kg), fumonisin B4 (FB4) (78%; range 5.1–1283.4 μg/kg), fumonisin B3 (FB3) (73%; range 10.3–948.3 μg/kg), fumonisin A2 (FA2) (66%; range 2.4–175.6 μg/kg) and fumonisin A1 (FA1) (54%; range 1.6–280.4 μg/kg).

**Figure 2:**
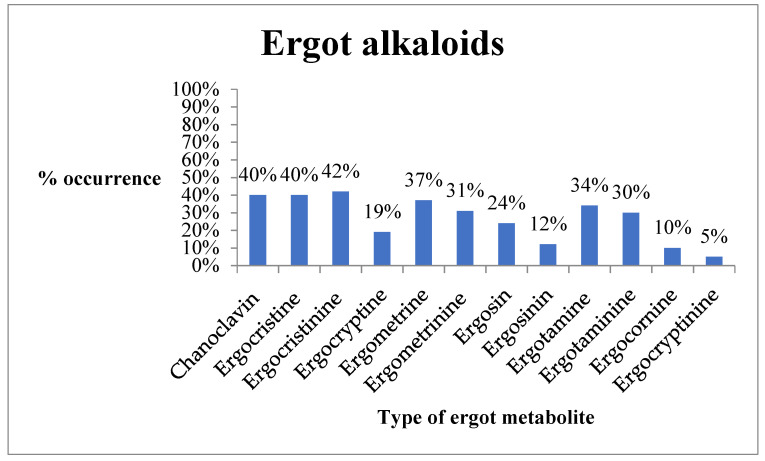
Occurrence of ergot alkaloids in feed and feed ingredients (*n* = 67) in Machakos (Kenya) in February and August 2019 period.

Ergocristinine (42%) was the most prevalent ergot alkaloid with other ergot alkaloids being; chanoclavin, ergocristine, ergocryptine, ergometrine, ergometrinine, ergosin, ergosinin, ergotamine, ergotaminine, ergocornine, and ergocryptinine (Figure 2).

DON-3-glucoside (DON-3-gluc), a mycotoxin conjugate (72%; range 2.0–63.4 μg/kg) had a high occurrence (98%) within the pool of samples that were contaminated with DON.

Occurrence levels of other secondary fungal, bacterial, plant and unspecified metabolites in the feed and feed ingredients are shown in the Supplementary Figures S1–S11.

The occurrence of common mycotoxins as per the type of feed is presented in Table 1. Dairy feed was contaminated with multiple mycotoxins in order of predominance; FUM (100%; range 52.4–2171.3 µg/kg), ZEN (100%; range 3.9–140.2 µg/kg), AFs (94%; range 1.5–318.5 µg/kg), DON (94%; range 66.1–567 µg/kg), NIV (94%; range 15.9–102.1 µg/kg), DON-3-gluc (88%; range 8.1–61.7 µg/kg), ergot alkaloids (63%, range 0.6–285.7 µg/kg), OTA (56%, range 2–24.3 µg/kg), T-2 (13%; range 2.7–4.4 µg/kg), and HT-2 (6%; mean 11.9 µg/kg).

AFB1 was the most prevalent of the AFs, occurring in 94% of the dairy feed samples (range 1.3–134 µg/kg), with 81.3% being above the East African Community (EAC) and EU Commission limit of 5 µg/kg. The overall mean of all the samples (13.5 µg/kg) was also above the limit. Other AFs were; AFG1 (88%; range 0.2–123 µg/kg), AFB2 (81%; range 0.91–22.1 µg/kg), AFG2 (44%; range 2.7–28.5 µg/kg) and AFM1 (38%; range 1.6–11 µg/kg). All other mycotoxins occurred at levels below the EU maximum guidance levels in dairy feeds

A similar occurrence pattern was observed in poultry feed samples, i.e., DON (range 28.2–1037 µg/kg), DON-3-gluc (range 3.8–45.7 µg/kg), FUM (range 63.7–2684.8µg/kg) and ZEN (range 5.2–873.4 µg/kg) occurred in all the poultry feed samples. Other frequently detected mycotoxins were; NIV (96%; range 12.1–105.5 µg/kg), AFs (93%; range 0.5–89 µg/kg), ergot alkaloids (81%; range 1.1–113.2 µg/kg. Ochratoxin A (OTA) (19%; range 2.5–10.6 µg/kg), T-2 (4%; range < LOD–5.2 µg/kg) and HT-2 (4%; range < LOD–13.8 µg/kg) had low occurrence in the poultry feeds.

Of all the poultry feed samples, 7.4% had levels above the EAC regulatory limit of 50 µg/kg for AFs in adult poultry feed. Aflatoxin B1 (93%; range 0.5–38.8 µg/kg) was the most prevalent of the AFs and 14.8% of the samples were contaminated with AFB1 above the EAC regulatory limit of 50 µg/kg for adult poultry feed.

Of the EU regulated mycotoxins, the highest level of FUM (11,658.7µg/kg) was reported in maize grains, however, the mean occurrence level of FUM was lower than for both dairy and poultry feed samples. HT-2 and T-2 were not detected in the feed ingredients. The levels of the other EU regulated mycotoxins were; ZEN (83%; range 0.3–910.4 µg/kg), ergot alkaloids (63%; range 0.4–24.8 µg/kg), DON (54%; range 22.2–996.1 µg/kg), AFs (29%; range 0.2–99.4 µg/kg), DON-3-gluc (29%; range 2–63.4 µg/kg) and OTA (8%; range 0.2–1.1 µg/kg). Similarly, AFB1 and AFG1 were the most prevalent AFs, occurring in 25% of all feed ingredients samples in the range of 0.9 to 49.8 µg/kg and 0.2 to 34.9 µg/kg, respectively.

Table 2 shows the occurrence of the common EU regulated mycotoxins in relation to the two sampling periods; February and August 2019. Overall, samples collected in August 2019 had a higher occurrence of mycotoxins as compared to samples collected in February 2019.

Other unregulated mycotoxins/metabolites were also detected. Seven *Alternaria* toxins; altersetin, AOH, AME, infectopyron, macrosporin, TEN, and TEA occurred at an incidence between 33–66%, with TEN being the most prevalent.

Of the other *Aspergillus* toxins aside from AFs, 3-nitropropionic acid (81%) was the most prevalent with the other toxins including aspochracin A, aspulvinone E, averantin, averufin, kojic acid, norsolorinic acid, O-methylsterigmatocystin, sterigmatocystin, viomellein, and versicolorin C occurring at between 9 and 67%.

Enniatins were the most prevalent depsipeptides, with enniatin B being the most prevalent at 73% and other Enniatins including enniatin B1, enniatin A1, enniatin A and enniatin B2 occurring at between 36 and 70%. Beauvericin was the least prevalent at 10%.

Contamination by *Fusarium* metabolites was between 16–99% with moniliformin being the most prevalent at 99%. Other *Fusarium* metabolites were; 15-hydroxyculmorin, acuminatum B, apicidin, antibiotic Y, aurofusarin, bikaverin, butenolid, culmorin, deoxyfusapyron, equisetin, fusaproliferin, fusapyron, fusaric acid, fusarinolic acid, monocerin, rubrofusarin, siccanol, W493, 5-hydroxyculmorin, and epiequisetin.

*Penicillium* toxins had an occurrence of between 6–99% with flavoglaucin and quinolactacin being the most prevalent at 99%. Others included; 7-hydroxypestalotin, andrastin A, citreohybridinol, citrinin, cyclopenin, cyclopenol, cyclopeptine, dechlorogriseofulvin, dihydrocitrinone, griseofulvin, mycophenolic acid, O-methylviridicatin, oxaline, pestalotin, questiomycin A, quinolactacin B, rugulovasine A, secalonic acid D, vermistatin, verrucofortine, verrucosidin, viridicatin, aurantiamin A, cycloaspeptide A, phenopyrrozin, and penicolinate.

Other fungal metabolites had an occurrence of between 3–96% and included apicidin D2, chrysogin, acuminatum C, ascochlorin, barceloneic acid, bassianolide, chlorocitreorosein, citreorosein, fungerin, ilicicolin E, LL-Z 1272e, mollicellin D, neoechinulin A, NP139, sclerotinin A, xanthotoxin, cercosporin, diplodiatoxin, and paspalin. Cytochalasins had a low occurrence with cytochalasin H (34%) and cytochalasin J (6%) being the only ones present.

Apart from fungal toxins, bacterial metabolites did occur at between 15–94% and included cyclo (L-Pro-L-Val), surfactin A, and surfactin B. Contamination by phytoestrogens was between 21–54% with abscisic acid, coumestrol, daidzein, daidzin, genistein, genistin, glycitin, and glycitein being the phytoestrogens detected. There was low contamination with other plant metabolites with lotaustralin being the most prevalent at 24% and linamarin (7%) and atropine (4%) being the other metabolites.

Other unspecific metabolites that contaminated the feeds included asperglaucide, asperphenamate, brevianamid F, cyclo(L-Pro-L-Tyr), emodin, endocrocin, fellutanine A, iso-rhodoptilometrin, N-benzoyl-phenylalanine, neoechinulin D, rugulusovin, skyrin, and tryptophol, and occurred at between 34–100%.

## 3. Discussion

This is the first study in Kenya to document the occurrence of mycotoxins, bacterial metabolites, and plant toxins using a multi-toxin detection method. The results document the occurrence of 153 different toxins and co-contamination of samples by more than one mycotoxin being common. The observed high occurrence of multiple mycotoxins in feed and feed ingredients corresponds to previous reports in Kenya [10,11,12,13,14]. However, most of the previous studies have focused on AFs with little done on other mycotoxins. The mixture of different *Fusarium* metabolites occurred in high frequency, which is in line with findings by Ezekiel et al. [20] and Streit et al. [18] who reported that *Fusarium* metabolites are the most abundant toxins in animal feeds. However, in our case, *Penicillium* toxins also did occur at a high frequency.

In Kenya, regulatory limits for mycotoxins in animal feed only exist for AFs [15], however, guidance limits have been set for DON, ergot alkaloids, FUM, OTA, and ZEN by other bodies such as the EU [15,19]. Of the regulated mycotoxins, ZEN was the most prevalent mycotoxin occurring in 94% of all the feed and feed ingredients (range; 0.3–910.4 µg/kg). This reported incidence and contamination level were higher than what has previously been reported in Kenya by Rodrigues et al. (56%, maximum; 167 µg/kg) [13]. In our study, the maximum level of ZEN reported in the dairy feed (140.2 µg/kg) was below the EU guidance level of 500 µg/kg, however, the maximum level (910.42 µg/kg) reported in feed raw ingredients was higher than the guidance limit. A similar higher occurrence has been reported in South Africa (96%, maximum; 123 µg/kg) 3 with lower incidences reported in Ghana (11%, maximum; 310 µg/kg) [13] and Nigeria (51%, maximum; 80 µg/kg) [13]. In dairy animals, high levels of ZEN have been reported to cause reduced feed intake, reduced milk yield, and reproductive disturbances [25], however, short-term exposure to this concentration of ZEN in the dairy feed may indicate ZEN may not cause acute problems but with 100% of the dairy feeds being contaminated this may cause chronic exposure and hence may affect fertility. Poultry are more tolerant of ZEN toxicity and currently, there is no guidance limit for ZEN in poultry feed in Kenya. This reported level of ZEN in poultry feed (100%, range; 5.2–873.4 µg/kg) may not singly have an acute impact on poultry health and productivity, however, recurrent exposure may have an impact on fertility.

Widespread FUM contamination of animal feed has been reported in Ghana [13], South Africa [3,13,26], Tanzania [27], Sudan [13], and Kenya [13]. In this study, 90% of all the samples had FUM with a mean of positives of 1051 µg/kg, and the maximum contamination level was detected in a maize sample (11,658.7 µg/kg). Similarly, high levels of FUM were reported by Nyangi et al. [27] in maize destined for animal feed in Tanzania. The levels of FUM reported were within the EU guidance levels for FUM in dairy (50 mg/kg) and poultry feed (20 mg/kg), however, due to co-occurrence with other mycotoxins, it may still cause a negative impact in poultry and dairy animal health due to synergistic or additive effects.

Type-B trichothecenes comprising of DON, the conjugate DON-3-glucoside, and NIV showed a significant incidence of contamination with a prevalence of 82%, 73%, and 72%, respectively. DON is the type B trichothecene that has received considerable worldwide interest, with the EU setting a guidance limit of 5000 µg/kg for complementary and alternative feedstuffs for both poultry and dairy animals. Pigs are the most sensitive species with ruminants being less sensitive with a drop in feed intake and a drop in milk yield being the major reported signs in dairy animals [15]. In poultry, high levels of DON have been reported to affect growth rate, feed conversion efficiency, and causing increased sensitivity to infectious diseases such as necrotic enteritis at levels below and approaching EU guidance level, and when combined with AFs causes additive toxicity. Despite the levels in this study being within the EU guidance limit, studies have shown that levels lower than the EU guidance level may affect metabolic, immunological, and physiological processes in animals [28,29]. Similarly, Makau et al. [11] in a study on contamination of dairy feeds (forages and concentrates) in Nakuru, Kenya, reported 63% of the samples had DON contamination with concentrates having a significantly higher mean level of contamination (86.95 µg/kg) but with all samples being below the EU guidance limit. The high occurrence of DON-3-gluc together with DON (98% co-occurrence), which is a modified mycotoxin that undergoes cleavage by lactic acid bacteria in the digestive tract of the mammals releasing DON, is of concern since it increases the exposure to DON in the contaminated feed [18,30]. Similar high co-occurrence of DON and DON-3-gluc has been reported by Streit et al. [18]. On the other hand, type A trichothecenes comprising of T-2 and HT-2 had a low occurrence (4% and 3% respectively). In poultry, T-2 is more toxic than HT-2, and at levels of 0.4 mg/kg and above causes oral lesions and decreases performance [29], while in dairy, aside from affecting milk yield and reproductive performance it also causes immunosuppression and gastroenteritis [15]. However, the highest level in this study was below the EU guidance level of 250 µg/kg.

The high incidence of total AFs (70%; range; 0.2–318.5 μg/kg) is in agreement with previous studies in Kenya by Okoth and Kola [12] on dairy feed (100% occurrence) and Rodrigues et al. [13] on animal feeds and raw materials (78% occurrence). AFB1 was the most prevalent of the AFs, occurring in 69% of the feed and raw material samples (range; 0.5–134 μg/kg). Similar findings have been reported by Senerwa et al. [31] in compounded dairy feeds in different regions of Kenya and by Makau et al. [11] in concentrates and forages in Nakuru, Kenya. Both dairy feed and poultry feed had a high occurrence of both total AFs and AFB1, however, the occurrence was at a higher level in the dairy feed (geomeans; 20.4 and 13.5 µg/kg, respectively) compared to poultry feed (geomeans; 6.2 and 4.7 µg/kg, respectively). This may be attributed to the raw materials used for the manufacture of dairy concentrates such as cottonseed cake and sunflower-seed cake that are very susceptible to high contamination by AFs [12,32]. In dairy animals, AFB1 at levels of 75 µg/kg–13 mg/kg have been reported to affect productivity, reproduction, cause hepatotoxicity and nephrotoxicity as well as causing immunosuppression [1,15]. Besides the animal health impact, there is a carry-over of AFB1 to milk as AFM1 and this poses a health hazard to humans since AFM1 is a human carcinogen [9]. Several studies in Kenya have reported an occurrence of between 39.7% and 100% of AFM1 in milk with the highest level being 4.63 µg/kg and mean occurrence levels of between 0.003 and 0.29 µg/kg. [5,7,14,32,33,34,35,36,37]. Between 10.4% and 64% of the positive milk samples in these studies exceeded the EU regulatory limit of 0.05 µg/kg for milk. This indicates exposure through contaminated feed. The carry-over of AFB1 to milk varies from less than 1% to 6.2% [38,39] with the level of carry-over usually determined by physiological and nutritional factors such as the animal species, individual animal variability, feeding regimens and type of diet, presence of other mycotoxins, stage of lactation, and actual milk production [15,24]. Therefore, with 81.3% of the samples exceeding the regulatory limit for both AFB1 (mean; 31.2 µg/kg) and AFs (mean; 61.5 µg/kg), it indicates a high risk of contamination of milk meant for human consumption and at levels above the EU (0.05 µg/kg) and East African Community (0.5 µg/kg) regulatory limit for AFM1 in milk, posing a health hazard to humans. In poultry, AFs are reported to cause decreased weight gain, poor feed efficiency, reduced egg production, hepatotoxicity, and immunosuppression [29]. Carry-over of AFs in poultry products occurs albeit at a smaller level than in milk [40,41]. Due to this high toxicity to both humans and animals and the carry-over to dairy and poultry products, EAC has set up regulatory limits for AFs and AFB1 in the dairy feed (10 µg/kg and 5 µg/kg, respectively) and adult poultry feed (20 µg/kg and 50 µg/kg respectively) [42]. In poultry, 14.8% and 7.4% exceeded the EAC regulatory limit for AFB1 and AFs, respectively, indicating a lower risk to animal and human health, however, the high incidence coupled with co-occurrence with other mycotoxins may increase the risk of chronic aflatoxins exposure.

Consumption of ergot-contaminated feed can have negative effects on feed intake, growth, and reproduction. Long term exposure of ergot alkaloids even of less than 2000 µg/kg depresses animal performance and causes intoxication [19]. In cattle, consumption of ergot contaminated feed affects animal growth (daily intake of 12.7 g) with chronic exposure reducing reproductive performance and causing abortion [19]. In comparison, poultry has a higher tolerance for ergot toxicity with levels as high as a 4 g/kg diet fed to 28-day old broilers having no effect [19]. However, long term exposure causes loss of appetite, increased thirst, diarrhea, vomiting, and weakness [43]. Currently, no regulatory limit for ergot alkaloids exists in Kenya with the EU setting a limit of 0.1 mg/kg in animal feed [19]. With an occurrence of 70% (range; 0.4–285.7 µg/kg), this shows a substantial amount of the feed was contaminated with ergot alkaloids. A total of 12 ergot alkaloids (chanoclavin, ergocristine, ergocristinine, ergocryptine, ergometrine, ergometrinine, ergosin, ergosinin, ergotamine, ergotaminine, ergocornine, and ergocryptinine) were reported, which was similar to what Ingenbleek et al. [44] reported in food processed from wheat in Benin, Cameroon, Mali, and Nigeria, except for chanoclavin.

OTA is rarely a problem in cattle due to the rumen’s ability to break down OTA into less toxic metabolites, with doses used in the experiment as high as 1.66 mg/kg body weight for 5 days not producing clinical disease [15]. With the highest level reported of 24.3 µg/kg, it can therefore be concluded that OTA is not a major problem in dairy cattle in Kenya, as previously concluded in a review by Kemboi et al. [15]. In poultry, high levels of OTA cause nephrotoxicity, hepatotoxicity, neurotoxicity, and immunosuppression, with the EU setting a limit of 100 µg/kg for complementary and complete poultry feed [45]. With an occurrence of 19% and the highest level of 10.6 µg/kg, it, therefore, indicates that these levels of OTA only may not be a major problem in poultry. Similarly, a previous study by Rodrigues et al. [13] also reported a low level of OTA (mean; 2 µg/kg) in animal feeds and raw material samples from Kenya.

Concerning the unregulated metabolites, data on occurrence and toxicity is rare in mammals. AME aside from being genotoxic has been shown to affect progesterone synthesis in pigs and postulated to have an impact on reproductive performance in other mammals [18]. TEA fed orally at 1.25–1.50 mg/kg body weight/day for 3 weeks causes a significant impact on the weight gain and causes lesions on chicken tissues [18]. Despite all samples having lower levels of TEA compared to the dose used in the experiment, one sample had levels of 7 mg/kg and this may have an impact on animal health.

Kojic acid and 3-nitropropionic acid that we reported in the feeds and feed ingredients are *Aspergillus* metabolites that have previously been shown to contaminate animal feeds [18,20]. Their toxicity to animals has not been demonstrated, but the presence of a high level of kojic acid indicates deterioration of the cereal component of the feed by *Aspergillus* since it is a metabolic byproduct produced during contamination of cereals [20].

Of the *Fusarium* metabolites reported, moniliformin that occurred in 99% of the samples and aurofusarin that occurred in 91% of the samples are toxic to animals. In chicken, aurofusarin affects egg quality by decreasing vitamins E, A, total carotenoid, lutein, and zeaxanthin concentrations, as well as affect the yolk color by increasing susceptibility to lipid peroxidation and the meat quality by decreasing protein and fat content [18,20,46]. In breeding chickens, feeding 26.4 mg/kg aurofusarin in feed compromises the immunity of the progeny18. Studies have shown high levels of moniliformin to be toxic to chicken [47], turkey [47], pigs [48], and sheep [49]. Broiler chickens fed feed contaminated with moniliform (50 mg/kg) to market age had a lower body weight gain, poor feed converting rate, and higher mortality [47]. Despite the low levels when compared to the toxic doses of moniliformin reported in these studies, combined with other toxins may be hazardous as a combination with AFs, DON and FB1 have been shown to cause additive effects in poultry and pigs [50,51,52].

The reported depsipeptides, enniatins, and beauvericin have been previously reported in feeds in South Africa [26], Nigeria [20], and in samples collected from Europe and America [18]. Beauvericin at levels of 2.5–12 mg/kg feed show low or no acute toxicity in broiler chicken and ducklings [53]. Little studies have shown the toxic effect of enniatins in livestock. A study by Fraeyman et al. [54] on chronic dietary intake of enniatin B in broiler chicken showed no major impact on intestinal morphometry and hepatic histology with a limited transfer to liver tissue. However, enniatin A has antibacterial, antifungal, herbicidal, insecticidal, and ionophore properties [18,20].

Emodin is a metabolite produced by *Aspergillus* as well as the plant, rhubarb root, at a frequency of 93% but at low concentration (range; 0.2–117 µg/kg) and has been experimentally shown to be toxic to chicken. One day old cockerels fed feed with 3.7 mg/kg body weight emodin had a loss of appetite, accumulation of fecal material with acute epidermal irritation around the cloaca, general debilitation, and mortality within 5 days of ingestion [18,55].

Phytoestrogens are non-steroidal phenolic plant compounds with a similar structure to estradiol and hence bind with estrogen receptors and may inhibit or promote estrogenic response. Soybean is the major source of phytoestrogens that can have adverse effects on animals [21,23]. Phytoestrogens may also compete with ZEN in binding to the estrogen receptors and thereby may counteract the estrogenic activity of ZEN [22]. The occurrence of the phytoestrogen in the study may lead to this interaction once consumed by an animal.

Cyclo (L-Pro-L-Val) was the most prevalent bacterial metabolite contaminating the feeds at a frequency of 94% with surfactant A and B also detected. With little studies done on the effects of these metabolites and some considered to be beneficial by being antibiotics, they may also lead to the development of antibiotic-resistant bacteria [20].

The high level of co-contamination of the feed and feed ingredients with the mycotoxins and/or metabolites is a concern. The majority of the samples (96%) were contaminated with more than two mycotoxins of animal health, public health, and international trade significance. This is similar to findings by Rodrigues et al. [13] on animal feeds and raw materials from Kenya but without quantification of the levels of co-occurrence of the mycotoxins. Makau et al. [11] on a study of dairy forages and concentrates in Nakuru, Kenya, also reported DON and AFs co-occurrence. Elsewhere in SSA, multiple mycotoxin occurrence in feeds has been reported in South Africa [3,26] and in human food in Benin, Cameroon, Mali, and Nigeria [20,44]. This co-occurrence may cause increased risk to animal and human health. A combination of mycotoxins at low concentrations that individually have no negative effect, may in combination negatively affect the animal [56]. In dairy, FUMs have been reported to increase the uptake of AFs and subsequently the carry-over to milk, and with 98% of feeds with AFs also having FUM there is an increased risk to animal health and food safety. OTA even at lower levels together with other mycotoxins including FB1, is the etiology of mycotoxic nephropathy in pigs and chickens reported in South Africa, Northern, and Eastern Europe [57,58]. In poultry, DON in addition to AFs shows additive toxicity [28]. Despite the individual effects of the other unregulated metabolites in poultry and dairy animals not being reported, studies have shown that some such as moniliformin have additive effects when combined with AFs, DON, and FB1. This occurrence of multiple mycotoxins in feed therefore presents a toxicological hazard to both dairy and poultry as well as to food safety even when the regulated mycotoxins occur at low levels. Apart from these, several mycotoxins such as AFs, OTA, and T-2 also cause immunosuppression in farm animals and this enhances the risk of the animals getting other diseases [18,24].

## 4. Conclusions

The study was conducted to explore the level of contamination of dairy feed, poultry feed, and feed raw ingredients in Machakos town, Kenya. This is the first multi-toxin study done in Kenya and 153 toxins, comprising mycotoxins, plant, and bacterial toxins were detected in the samples. This information provides much-needed input that is useful when coming up with mycotoxin mitigation strategies. However, it should be noted that not all of the 153 toxins represent a hazard to animal health, but toxicological interactions may enhance the toxicity of other regulated mycotoxins and hence representing increased animal health and public health hazard. It should also be noted that the production of mycotoxins is related to environmental conditions and this may contribute to seasonal as well as year to year variation of mycotoxins contamination [59]. Machakos county where the samples were collected is located in the lower midland agro-ecological zones and receives between 200 and 1200 mm of rainfall yearly with frequent droughts resulting in crop failures [60], this cause stress to plants and may significantly contribute to a higher contamination level in feed during such seasons. Other factors such as poor storage, damage to the grains caused by rodents and pests, and abiotic factors such as pH of feed and moisture content can also cause variation between batches of feeds in the same season [59]. Within the same batch, heterogeneous contamination of samples by mycotoxins may also occur causing high variations in observed levels of mycotoxins in the sample. Of the regulated mycotoxins, AFs represent the major challenge to both poultry and dairy due to the high numbers above the regulatory limit with the more potent AFB1 being the most prevalent. These possess a food safety challenge due to the carry-over of AFB1 from feed to milk and poultry products. Other mycotoxins, especially Fusarium mycotoxins, occurred at high incidences but were within the guidance limit. There is therefore a need for stronger enforcement of regulations to protect animal health and productivity and ensure food safety.

## 5. Materials and Methods

### 5.1. Study Site

The study was undertaken in Machakos town in Machakos County, Kenya. Machakos was purposively selected due to previous studies indicating a high prevalence of AFs in food and feed. A total of 67 samples (1 kg each) sampled from the top, middle, and bottom part of each bag comprising compounded dairy and poultry feed and feed ingredients were collected from animal feed retail shops (Agrovets) and market stalls dealing with cereal grains. The sampling was done by the individual attendants in each of the shops. Forty-seven samples comprising of 7 dairy feed, 16 poultry feed, and 24 feed ingredients (maize, soybean meal, and cottonseed cake) were collected in February 2019, while in August 2019, 20 samples were collected comprising 9 dairy feed and 11 poultry feed but no feed ingredients.

### 5.2. Sample Preparation

The samples were milled to fine uniform particle size using a warring blender (Waring Products DIV., Torrington, CT) and subsequently mixed before a subsample was collected for analysis. The samples were stored at −20 °C until analysis.

### 5.3. Analytical Method

All samples were analyzed for the presence and levels of mycotoxins and other secondary metabolites by LC-MS/MS as described by Sulyok et al. [61]. This fully validated method enables the accurate quantification of more than 500 secondary (toxic) metabolites of plants, bacteria, and fungi including all relevant mycotoxins. In cooperation with the University of Natural Resources and Life Sciences, Vienna (BOKU/IFA-Tulln) this method was introduced into the market as Spectrum 380^®^ by BIOMIN in the year 2014. Briefly, 5 g of finely ground sample was weighed into a 250 mL Erlenmeyer flask and extracted for 90 min using 20 mL of acetonitrile/water/acetic acid in the ration of 79/20/1 (*v*/*v*/*v*). The samples were shaken for 90 min using a GFL 3017 rotary shaker (GFL, Burgwedel, Germany) and subsequently centrifuged for 2 min at 3000 rpm on a GS-6 centrifuge (Beckman Coulter Inc., Brea, CA, USA). The extracts were transferred into glass vials using Pasteur pipettes, diluted 1:1 with acetonitrile/water/acetic acid (79/20/1), and subsequently analyzed by injecting 5 μL into the LC-MS/MS system (Applied Biosystems, Foster City, CA, USA).

Chromatographic separation was achieved by binary gradient elution of mobile phase A (methanol/water/acetic acid, 10/89/1, *v*/*v*/*v*) and mobile phase B (methanol/water/acetic, 97/2/1, *v*/*v*/*v*) with both containing 5 mM ammonium acetate and pumped at a flow rate of 1000 μL/min on a Gemini C18-column, 150 × 4.6 mm i.d., 5 μm particle size, equipped with a C18 security guard cartridge, 4 × 3 mm i.d. (both Phenomenex, Torrance, CA, USA). The elution consisted of an initial 2 min at 100% mobile phase A and a linear increase of mobile phase B to 50% within 3 min and further to 100% within 9 min, followed by a hold-time of 4 min at 100% mobile phase B and a 2.5 min column re-equilibration at 100% mobile phase A. The injection volume of both the samples and the mycotoxin standard solutions was 5 μL. Identification and quantification of each mycotoxin were performed in the Selected Reaction Monitoring (SRM) mode using a QTrap 5500 LC-MS/MS system (Applied Biosystems, Foster City, CA, USA). External calibration was done using multi-analyte working solutions prepared by mixing different mycotoxins working solutions and mobile phase A.

### 5.4. Data Management and Analysis

Data was entered in Microsoft^®^ excel and analysis was done using R version 3.6.1. The occurrence, the geometric and arithmetic means, and the range of each mycotoxin or metabolite was calculated. The arithmetic mean was calculated for the positive samples and the geometric mean for all the samples. For calculation of geometric mean, half the value of the limit of detection (LOD) for samples with levels below the LOD was used. Comparison of the mycotoxin or metabolite occurrence and level was done between the dairy feed, poultry feed, and feed ingredients as well as between the two sampling periods.

## Figures and Tables

**Table 1:** The occurrence of common EU regulated mycotoxins in feed and feed ingredients in Machakos (Kenya) in February and August 2019 period.

	All Feed and Feed Ingredients (*n* = 67)					Dairy Feed (*n* = 16)				Poultry Feed (*n* = 27)				**Feed Ingredients (Cottonseed, Soybean Meal, Maize) (*n* = 24)**			
	LOD (µg/kg)	% POSITIVE	GEOMEAN (µg/kg)	RANGE (µg/kg)	MEAN ± SD (µg/kg)	% POSITIVE	GEOMEAN (µg/kg)	RANGE (µg/kg)	MEAN ± SD (µg/kg)	% POSITIVE	GEOMEAN (µg/kg)	RANGE (µg/kg)	MEAN ± SD (µg/kg)	% POSITIVE	GEOMEAN (µg/kg)	RANGE (µg/kg)	MEAN ± SD (µg/kg)
**AFB1**	0.2	69	2.3	0.5–134	18.3 ± 23.7	94	13.5	1.3–134	31.2 ± 34	93	4.7	0.5–38.8	10.2 ± 10	25	0.3	0.9–49.8	19.7 ± 17.2
**AFB2**	0.06	45	0.2	0.4–22.1	3.4 ± 4.3	81	1.4	0.91–22.1	5.1 ± 5.8	48	0.2	0.4–4.4	1.7 ± 1	17	0.1	1.2–7	3.4 ± 2.1
**AFG1**	0.2	58	1.1	0.2–123	13.7 ± 20.9	88	5.6	0.2–123	21.7 ± 29.8	70	1.2	0.6–41.7	6.7 ± 9.8	25	0.3	0.2–34.9	17.1 ± 11.8
**AFG2**	0.5	31	0.6	0.5–28.5	5.1 ± 6	44	1	2.7–28.5	8.8 ± 8.6	33	0.5	0.5–6.4	2.5 ± 2	21	0.4	1.6–9.6	4.6 ± 2.9
**AFM1**	0.1	22	0.1	0.4–11	2.6 ± 2.8	38	0.2	1.6–11	3.7 ± 3.3	15	0.1	0.4–0.6	0.5 ± 0.1	21	0.1	0.5–6.9	2.9 ± 2.3
**AFs**	0.1	70	2.3	0.2–318.5	34.5 ± 51.5	94	20.4	1.5–318.5	61.5 ± 76.6	93	6.2	0.5–89	17.2 ± 20.5	29	0.2	0.2–99.4	38.9 ± 33.1
**DON**	0.4	82	64.4	22.2–1037	317.5 ± 224.9	94	195.4	66.1–567	359.4 ± 159.1	100	271.3	28.2–1037	329.1 ± 203.2	54	6.1	22.2–996.1	244.9 ± 302.5
**DON-3-gluc**	1	72	5.7	2.0–63.4	18.5 ± 13.7	88	11.6	8.1–61.7	22.1 ± 14.8	100	13.9	3.8–45.7	16.4 ± 9.7	29	1.2	2–63.4	19.4 ± 21
**NIV**	0.02	70	0.9	0.4–285.7	26.1 ± 29.2	94	32.8	15.9–102.1	51.1 ± 26.8	96	31.9	12.1–105.5	43.2 ± 22.5	33	1.9	10–144	50.2 ± 46
**FA1**	0.6	54	2.7	1.6–280.4	35.3 ± 51.2	38	1.7	13.8–83.2	39 ± 23.8	52	2	3.3–29.2	14.2 ± 8.1	67	5.3	1.6–280.4	52.2 ± 70.4
**FA2**	0.6	66	4.7	2.4–175.6	31.2 ± 34.2	75	7.7	4.7–87.2	31.9 ± 24.9	74	5.9	2.4–103.1	24.5 ± 26	50	2.6	5.7–175.6	41.6 ± 48.6
**FB1**	2	90	198.5	32.4–8345.6	742 ± 1223.6	100	311.2	52.4–1494	487.9 ± 407.1	100	305.5	38.4–1926	431.4 ± 387.2	71	90.5	32.4–8345.6	1474.4 ± 2034.7
**FB2**	2	85	72.8	16.7–3313.1	325.4 ± 554.4	94	91	26.6–677.3	175.5 ± 158.3	96	102.2	23.5–728.8	172.9 ± 156.7	67	42.9	16.7–3313.1	713.8 ± 906.5
**FB3**	6	73	32.6	10.3–948.3	136.3 ± 197.1	63	22.1	26.4–124.3	79.8 ± 30.6	85	36.7	20.4–243	70.8 ± 51.8	67	37	10.3–948.3	265.8 ± 299.4
**FB4**	6	78	55.8	5.1–1283.4	127.2 ± 212.6	75	38.8	6.7–124.8	54.2 ± 34.9	89	41.3	5.5–387.8	73.7 ± 95.1	67	115.1	5.1–1283.4	262.3 ± 325.4
**FUM**	0.6	90	264.6	32.4–11, 658.7	1051.1 ± 1722.4	100	414.9	52.4–2171.3	652.4 ± 559.8	100	420	63.7–2684.8	597.9 ± 541.5	71	116.6	32.4–11,658.7	2146.2 ± 2612.9
**OTA**	1	3	0.55	11.9–13.8	12.9 ± 5.6	56	1.2	2–24.3	5.6 ± 6.8	19	0.4	2.5–10.6	4.8 ± 3	8	0.3	0.2–1.1	0.6 ± 0.4
**Ergot**	0.4	73	11.9	9.9–144	46.8 ± 49.9	63	1.7	0.6–285.7	56.9 ± 88	81	3.1	1.1–113.2	26 ± 32.4	63	0.2	0.4–24.8	5.9 ± 7.4
**HT-2**	0.5	24	0.5	1.1–24.3	4.8 ± 1	6	0.6	11.9–11.9	11.9 ± 0	4	0.6	13.8–13.8	13.8 ± 0	ND	ND	ND	ND
**T-2**	0.7	4	0.4	2.7–5.2	4.1 ± 1	13	0.5	2.7–4.4	3.5 ± 0.8	4	0.4	5.2–5.2	5.2 ± 0	ND	ND	ND	ND
**ZEN**	0.2	94	18.1	0.3–910.4	81.3 ± 165.7	100	19.9	3.9–140.2	35.2 ± 40.7	100	56.1	5.2–873.4	103.4 ± 178.6	83	4.8	0.3–910.4	71.3 ± 196.7

AFs—Total aflatoxins, AFB1 –Aflatoxin B1, AFB2—Aflatoxin B2, AFG1—Aflatoxin G1, AFG2—Aflatoxin G2, AFM1—Aflatoxin M1, DON—Deoxynivalenol, DON-3-gluc—DON-3-glucoside, Ergot—Ergot alkaloids, FA1—Fumonisin A1, FA2—Fumonisin A2, FB1—Fumonisin B1, FB2—Fumonisin B2, FB3—Fumonisin B3, FB4—Fumonisin B4, FUM—Fumonisin B1 + Fumonisin B2, HT-2—HT-2 toxin, Geomean—Geometric mean of all the samples; *n*—number; Mean—mean of only the positives; LOD—Limit of detection; OTA—Ochratoxin A, Range—the range of positives; SD—standard deviation; T-2–T-2 toxin, ZEN—Zearalenone.

**Table 2:** The occurrence of common EU regulated mycotoxins as per sampling period.

	February 2019 (*n* = 47)			August 2019 (*n* = 20)		
	% POSITIVE	GEOMEAN (µg/kg)	RANGE (µg/kg)	% POSITIVE	GEOMEAN (µg/kg)	RANGE (µg/kg)
AFB1	60	1.4	0.5–134	90	7.5	3.4–38.8
AFB2	38	0.2	0.4–22.1	60	0.4	0.9–5.3
AFG1	47	0.7	0.2–123	85	3.2	1.6–41.7
AFG2	34	0.6	0.5–28.5	25	0.5	0.6–12.5
AFM1	32	0.2	0.4–11	ND	ND	ND
AFs	62	1.2	0.2–318.5	90	10	3.4–89.0
DON	77	41.6	22.2–1037	95	179.9	28.2–743.3
DON-3-gluc	62	3.7	2–63.4	95	14.5	3.8–61.9
NIV	66	7.9	9.9–144	95	30.2	15.9–102.1
FA1	72	5.7	1.6–280.4	10	0.5	21.7–43.9
FA2	57	3.2	5.3–175.6	85	11.8	2.4–103.1
FB1	85	145.9	32.4–8345.6	100	409	69.7–1926
FB2	81	56.5	16.7–3313.1	95	132.3	28.3–728.8
FB3	75	35.3	10.3–948.3	70	27	20.5–172.7
FB4	81	49.5	5.1–1283.4	70	77.3	7.6–387.8
FUM	85	192	32.4–11,658.7	100	562.4	98–2654.8
OTA	6	0.3	0.2–24.3	65	1.4	1.9–10.6
Ergot	77	1.1	0.4–154.5	55	0.5	0.6–285.7
HT-2	ND	ND	ND	10	0.7	11.9–13.8
T-2	2	0.4	2.7	10	0.5	4.4–5.2
ZEN	92	14.4	0.3–910.4	100	30.8	4.2–131

AFs—Total aflatoxins, AFB1—Aflatoxin B1, AFB2—Aflatoxin B2, AFG1—Aflatoxin G1, AFG2—Aflatoxin G2, AFM1—Aflatoxin M1, DON—Deoxynivalenol, DON-3-gluc—DON-3-glucoside, Ergot—Ergot alkaloids, FA1—Fumonisin A1, FA2—Fumonisin A2, FB1—Fumonisin B1, FB2—Fumonisin B2, FB3—Fumonisin B3, FB4—Fumonisin B4, FUM—Fumonisin B1 + Fumonisin B2, HT-2—HT-2 toxin, Geomean—Geometric mean of all the samples; *n*—number; ND—Not detected; OTA—Ochratoxin A; % Positive—above Limit of detection; Range—the range of positives; T-2–T-2 toxin, ZEN—Zearalenone.

## Supplementary Materials

The following are available online at https://www.mdpi.com/2072-6651/12/12/762/s1, Figure S1: Alternaria toxins, Figure S2: Aspergillus toxins, Figure S3: Bacterial metabolites, Figure S4: Cytochalasins, Figure S5: Depsipeptides, Figure S6: Fusarium metabolites, Figure S7: Metabolites from other fungi, Figure S8: Penicillium toxins, Figure S9: Phytoestrogens, Figure S10: Plant metabolites, Figure S11: Unspecific metabolites, Table S1: Co-occurrence of some common mycotoxins in dairy feed, poultry feed, and raw materials in Machakos, Kenya
