# Supplementary material for: Multi-Mycotoxin Occurrence in Dairy Cattle and Poultry Feeds and Feed Ingredients from Machakos Town, Kenya
Source: Toxins (Basel). 2020 Dec 3;12(12):762. doi: 10.3390/toxins12120762 (PMC7761711; doi:10.3390/toxins12120762)
Supplement: Supplementary file 1 [file toxins-12-00762-s001.pdf]

Supplementary Materials: Multi-Mycotoxin Occurrence in Dairy Cattle and Poultry Feeds and Feed Ingredients from Machakos Town, Kenya

David Chebutia Kemboi, Phillis E. Ochieng, Gunther Antonissen, Siska Croubels, Marie-Louise Scippo, Sheila Okoth, Erastus K. Kangethe, Johannes Faas, Barbara Doupovec, Johanna F. Lindahl and James K. Gathumbi

Occurrence level of other secondary fungal, bacterial, plant and unspecified metabolites in the feed and feed ingredients are shown in the figures below.

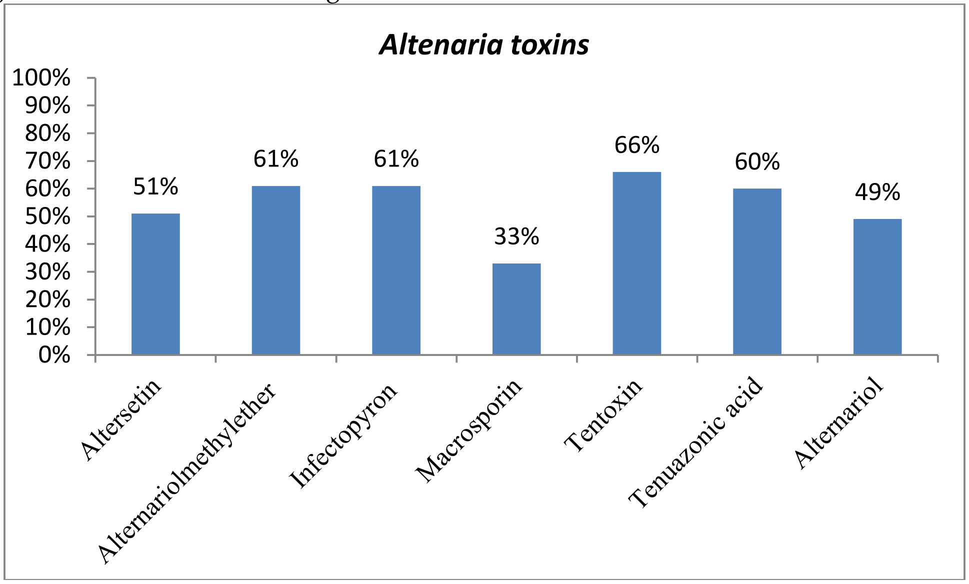

Figure S1. *Alternaria* toxins.

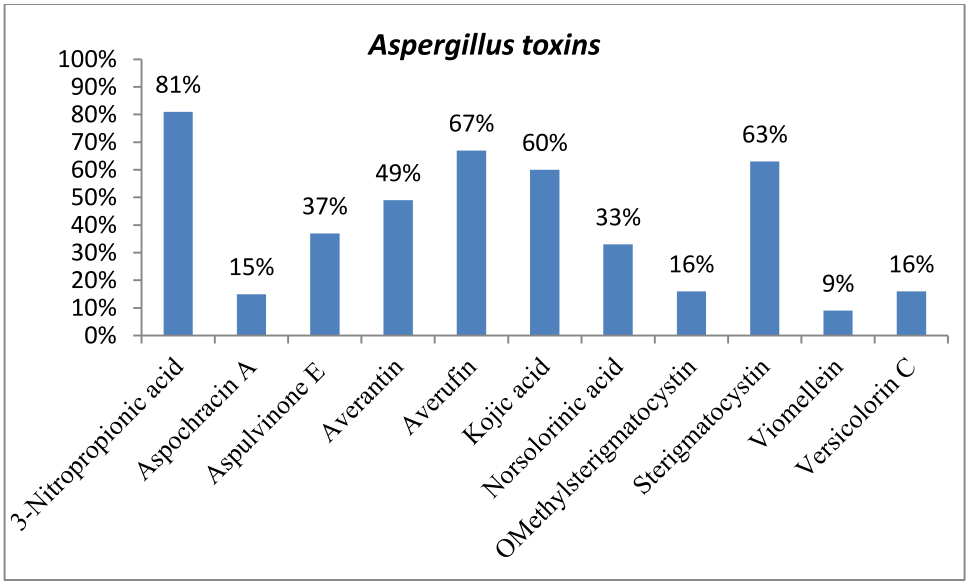

Figure S2. *Aspergillus* toxins.

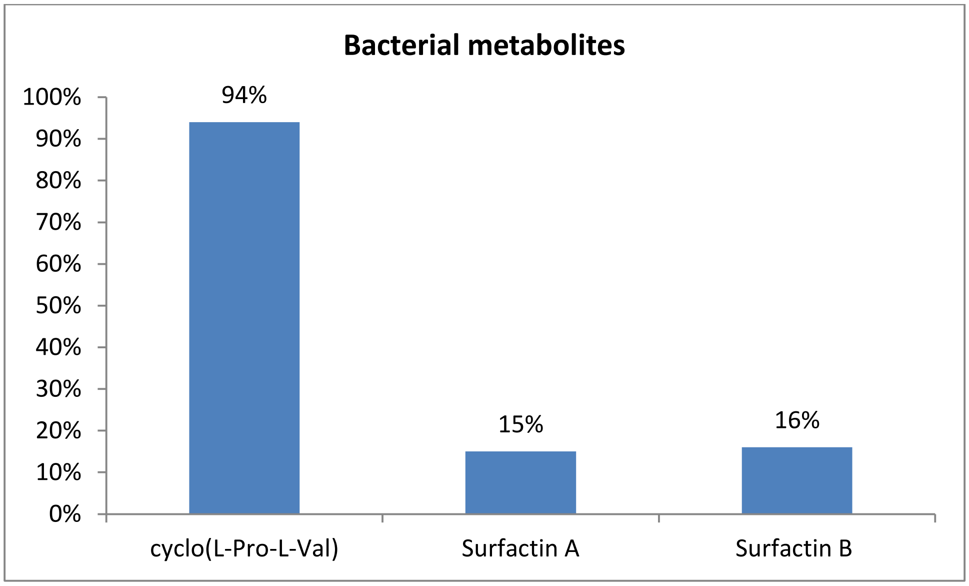

Figure S3. Bacterial metabolites.

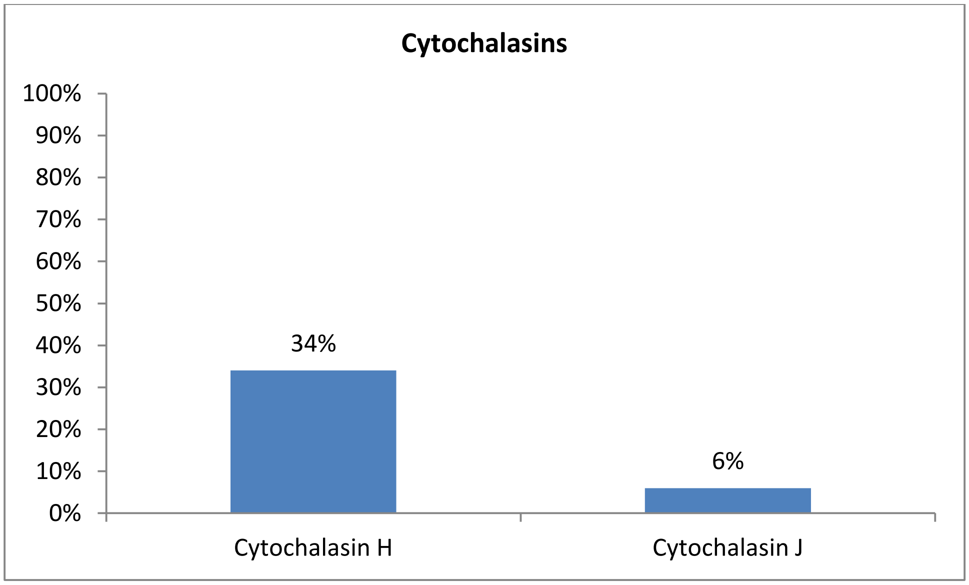

Figure S4. Cytochalasins.

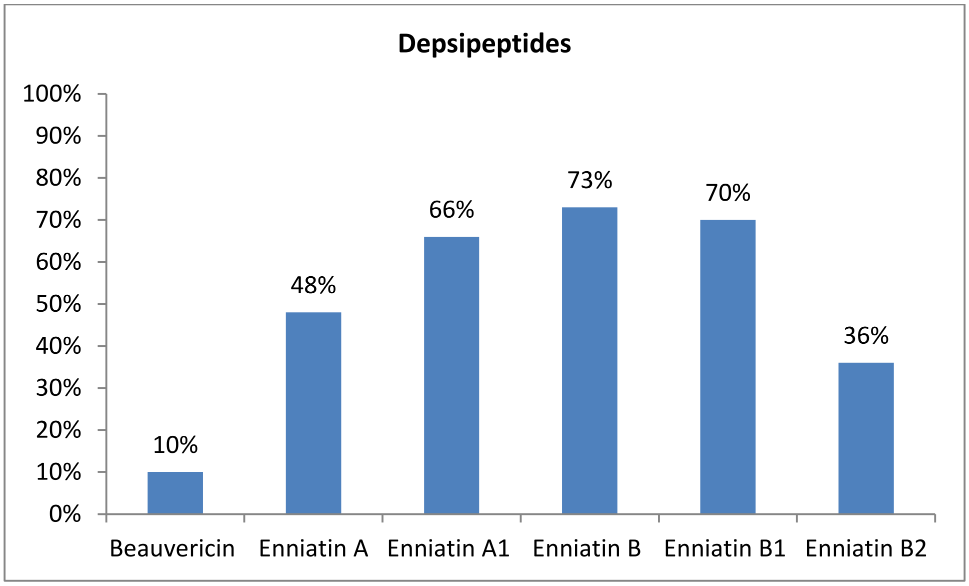

Figure S5. Depsipeptides.

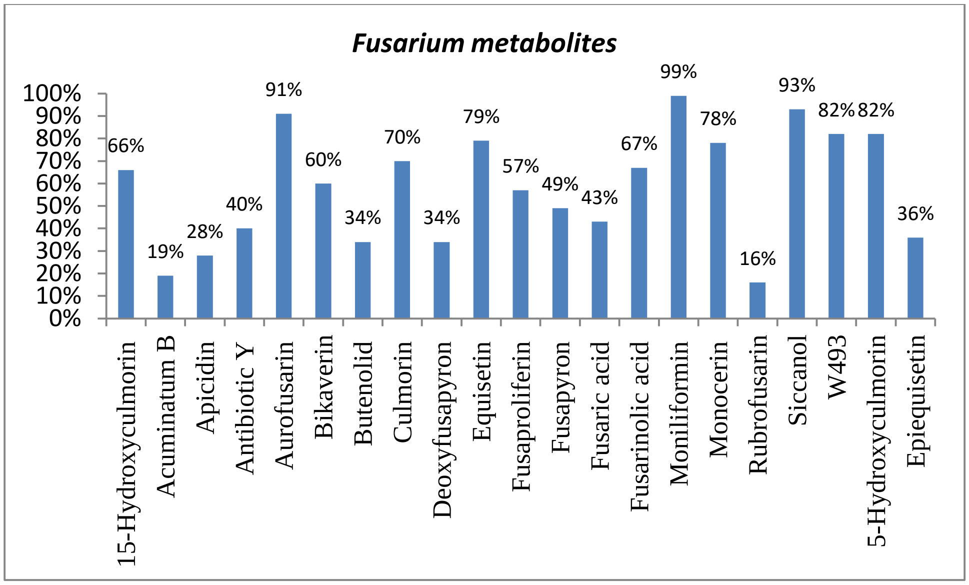

Figure S6. *Fusarium* metabolites.

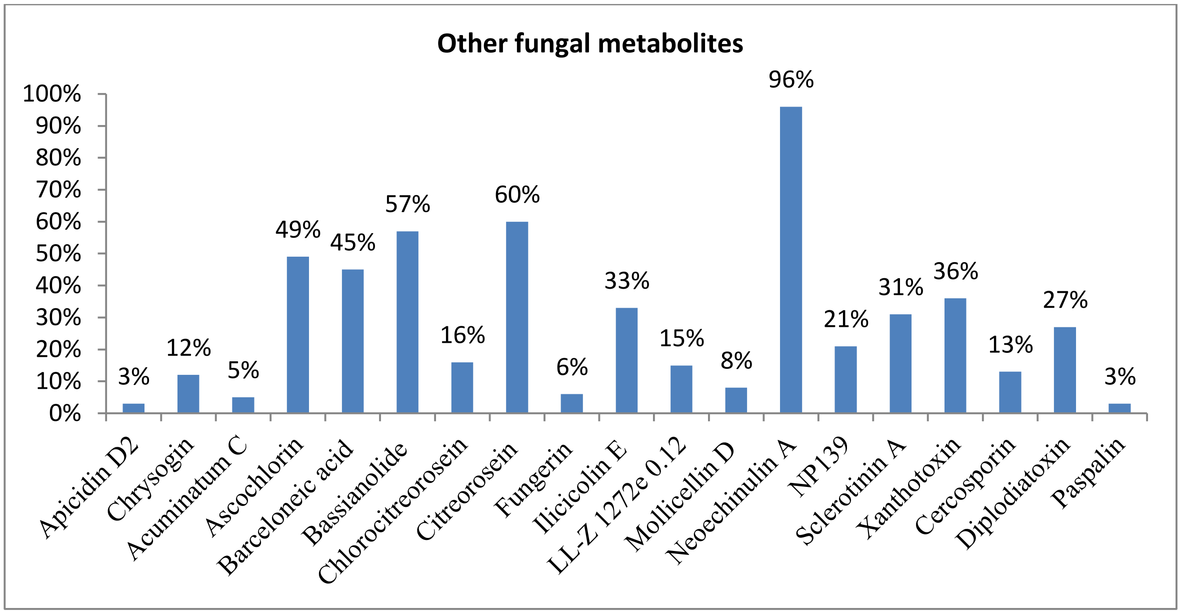

Figure S7. Metabolites from other fungi.

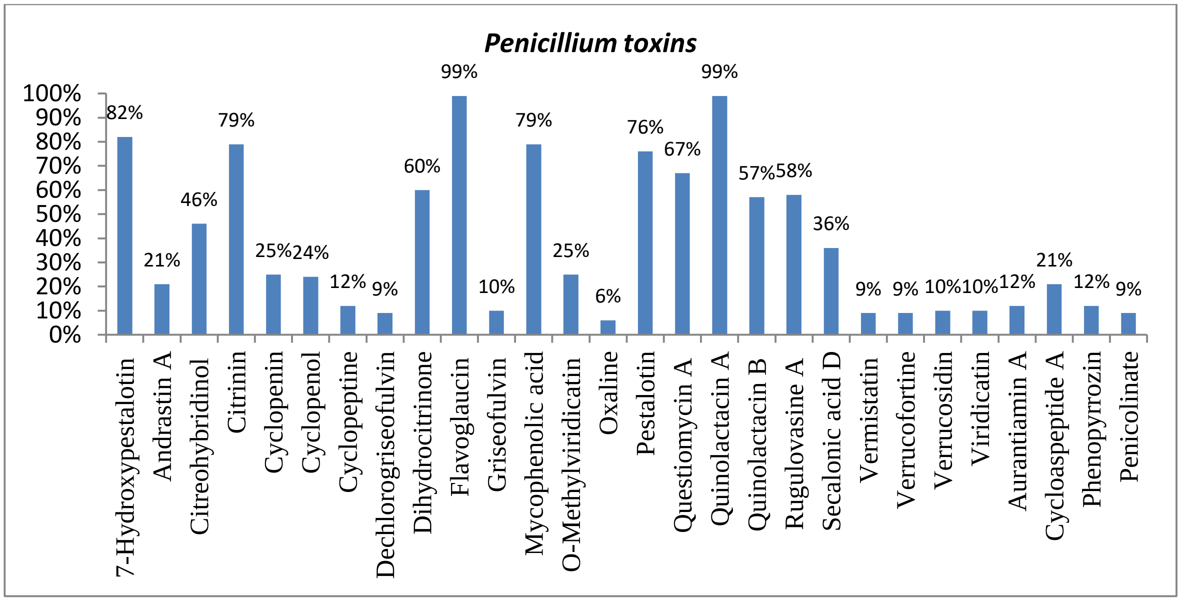

Figure S8. *Penicillium* toxins.

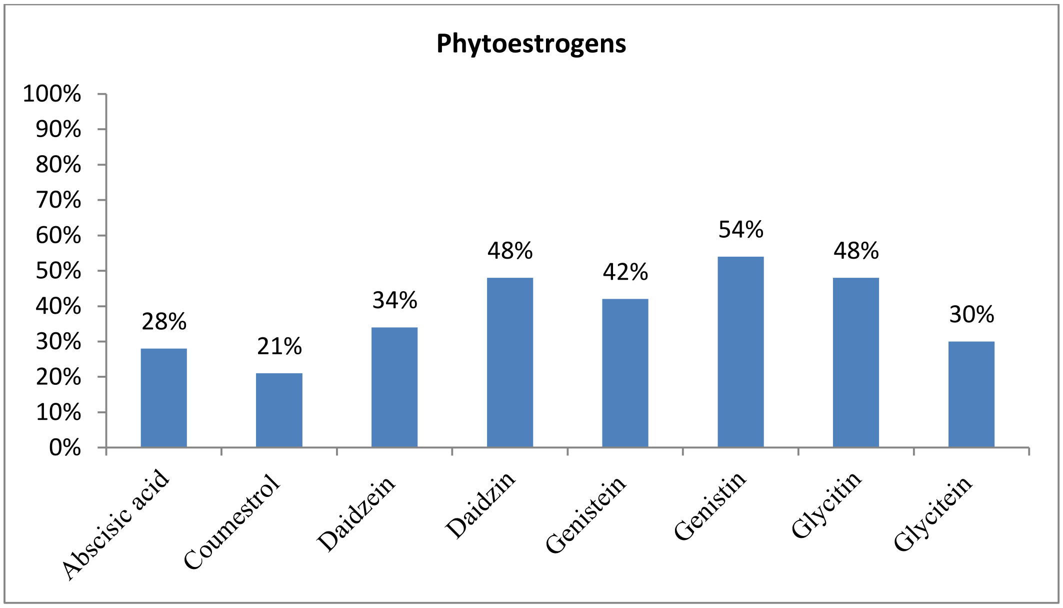

Figure S9. Phytoestrogens.

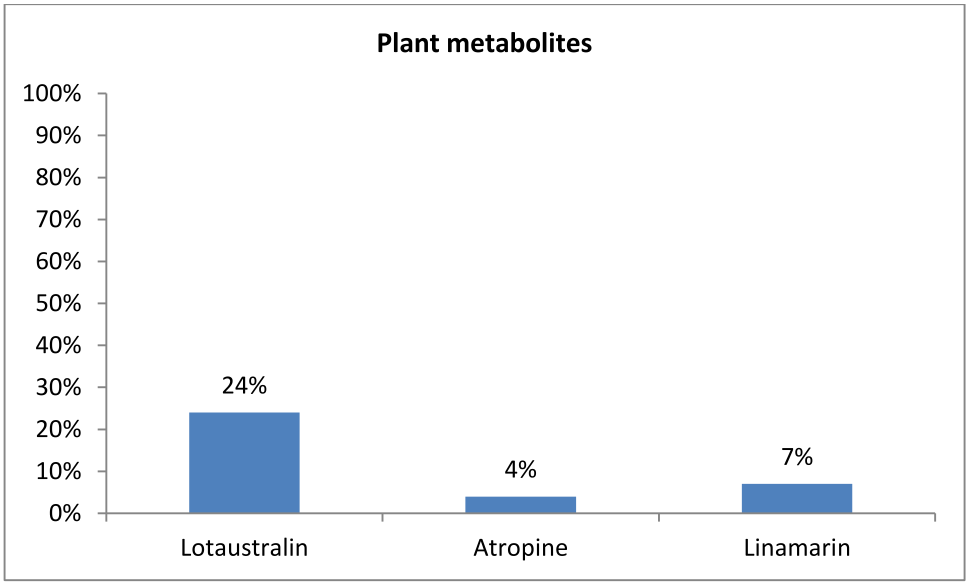

Figure S10. Plant metabolites.

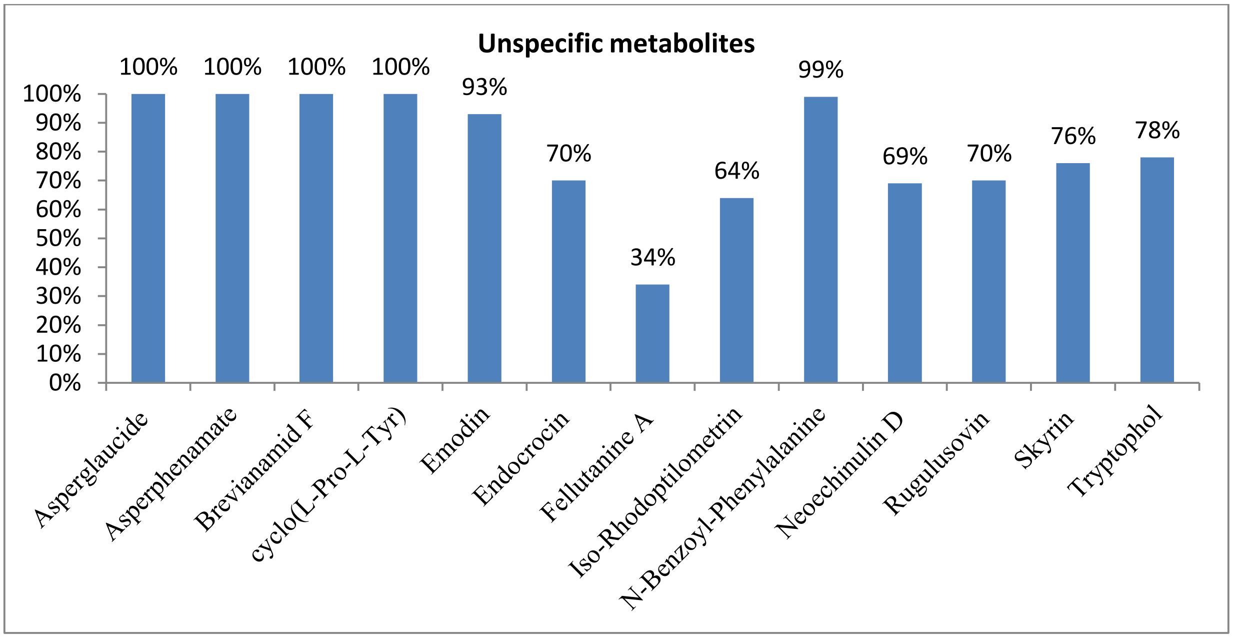

Figure S11. Unspecific metabolites.

Table S1. Co-occurrence of some common mycotoxins in dairy feed, poultry feed and raw materials in Machakos, Kenya.

Sample	AF	DON	FUM	OTA	Ergot	HT-2	T-2	ZEN	DON-3	NIV	Total
Fish meal	√							√			2
Chick mash	√	√	√		√			√	√	√	7
Layer mash	√	√	√		√			√	√	√	7
Layer mash	√	√	√		√			√	√	√	7
Kienyeji mash	√	√	√		√			√	√	√	7
Layer mash	√	√	√		√			√	√	√	7
Chick mash	√	√	√		√			√	√	√	7
Broiler finisher	√	√	√		√			√	√	√	7
Grower mash	√	√	√		√			√	√	√	7
Grower mash		√	√		√			√	√	√	6
Cotton seed mash	√		√					√		√	4
Grower mash	√	√	√		√			√	√	√	7
Grower mash	√	√	√					√	√	√	6
Chick mash	√	√	√		√			√	√	√	7
Dairy meal	√	√	√		√		√	√	√	√	8
Dairy meal	√	√	√		√			√	√	√	7
Dairy meal	√	√	√		√			√	√	√	7
Dairy meal	√	√	√					√	√	√	6
Chick mash	√	√	√		√			√	√	√	7
Maize germ	√	√	√		√			√	√	√	7
Layer mash	√	√	√		√			√	√	√	7
Layer mash	√	√	√		√			√	√	√	7
Cotton seed mash	√		√					√		√	4
Dairy meal	√	√	√	√	√			√			6
Dairy meal	√	√	√		√			√	√	√	7
Layer mash	√	√	√		√			√	√	√	7
Dairy meal	√	√	√		√			√	√	√	7
Soya meal	√	√	√		√			√	√	√	7
Maize grain			√		√			√	√		4
Maize grain		√	√		√			√			4
Maize germ		√	√		√			√	√	√	6
Maize grain			√		√						2

Maize grain			√								1
Maize germ	√	√	√		√			√	√	√	7
Maize grain		√	√	√	√			√			5
Maize grain		√	√					√		√	4
Maize grain		√			√			√			3
Maize grain								√			1
Maize grain					√						1
Maize grain		√	√		√			√	√		5
Maize grain		√			√			√	√		4
Maize grain	√		√		√			√			4
Maize grain		√						√			2
Maize grain		√		√	√			√			4
Maize grain		√	√								2
Maize grain			√					√			2
Maize grain			√		√			√		√	4
Dairy meal	√	√	√	√				√		√	6
Chick mash	√	√	√	√	√			√	√	√	8
Dairy meal	√	√	√	√				√	√	√	7
Dairy meal	√	√	√	√				√	√	√	7
Dairy meal	√	√	√	√	√			√	√	√	8
Dairy meal	√		√	√	√	√		√	√	√	8
Chick mash		√	√		√			√	√	√	6
Grower mash	√	√	√					√	√	√	6
Grower mash	√	√	√	√				√	√	√	7
Dairy meal	√	√	√	√			√	√	√	√	8
Dairy meal		√	√		√			√	√	√	6
Dairy meal	√	√	√	√				√	√	√	7
Dairy meal	√	√	√	√	√			√	√	√	8
Chick mash	√	√	√	√	√			√	√	√	8
Chick mash	√	√	√	√	√			√	√	√	8
Layer mash	√	√	√		√			√	√	√	7
Layer mash	√	√	√		√			√	√	√	7
Grower mash	√	√	√		√			√	√	√	7
Grower mash	√	√	√	√				√	√		6
Chick mash	√	√	√			√	√	√	√	√	8

AF—Total Aflatoxins; DON—Deoxynivanelol ; DON-3—DON-3-glucoside ; FUM—Total fumonisins ; Ergot—Sum of Ergot alkaloids ; HT-2—HT-2 toxin ; NIV—Nivalenol; OTA—Ochratoxin A ; T-2—T-2 toxin; ZEN—Zearalenone; √—Present.
